# A cross-lagged analysis of the relationship between adolescents' aggressive behavior, parent-child relationships, and teacher caring behaviors

**DOI:** 10.3389/fpsyg.2025.1605677

**Published:** 2025-10-22

**Authors:** Na Li, Tianpei Li, Lumin Liu, Duo Zhang

**Affiliations:** ^1^School of Humanities and Social Sciences, North University of China, Taiyuan, Shanxi, China; ^2^School of Physical Education, Jeonbuk National University, Jeonju, Republic of Korea

**Keywords:** aggressive behavior, parent-child relationships, teacher caring behaviors, adolescents, cross-lagged analysis

## Abstract

**Background:**

Aggressive behavior in secondary school students significantly affects their mental health, academic performance, and social adjustment. Parent-child relationships and caring teacher behaviors are recognized as key influences. However, most existing studies employ a cross-sectional design, limiting their ability to reveal dynamic causal relationships among variables.

**Purpose:**

This study aimed to examine the longitudinal mechanisms underlying the interactions between parent-child relationships, caring teacher behaviors, and aggression in middle school students using a cross-lagged model.

**Methods:**

Data were collected from 824 junior and senior high school students in Shandong Province using a longitudinal design, with a two-stage follow-up survey (one semester apart) employing the Aggression Behavior Scale, the Parent-Child Relationship Scale, and the Teacher Caring Behavior Scale. Correlation analyses and cross-lagged modeling tests were performed using SPSS and Amos, with measures taken to control for common method bias.

**Results:**

(1) T1 parent-child relationship significantly negatively predicted T2 aggressive behavior (β = −0.231, *p* < 0.001), but the reverse path was not significant. (2) T1 teacher caring behaviors significantly negatively predicted T2 aggressive behaviors (β = −0.142, *p* < 0.001), and T1 parent-child relationships positively influenced T2 teacher caring behaviors (β = 0.097, *p* = 0.009). (3) Aggressive behavior demonstrated temporal stability (β = 0.114, *p* = 0.002).

**Conclusion:**

Both parent-child relationships and caring teacher behaviors independently mitigate aggression in middle school students, with parent-child relationships potentially exerting an indirect effect by enhancing caring teacher behaviors. These findings highlight the significance of collaborative family-school interventions and offer a theoretical foundation for preventing adolescent behavioral problems.

## Introduction

### Background of the study

Aggression in middle school students has garnered significant research attention due to its far-reaching impact on mental health, social adjustment, the school environment, and broader societal stability. Aggressive behavior typically involves physical or psychological harm to others, encompassing physical violence, verbal aggression, and social ostracism (Hay et al., 2021; Tremblay, 2000). Studies indicate that adolescent aggression not only correlates with lower academic achievement and poorer peer relationships but also serves as a potential predictor of future antisocial behavior and criminal tendencies (Loeber and Hay, 1997; Naseem and Munaf, 2022).

First, investigating aggression in middle school students facilitates an understanding of its developmental mechanisms and underlying causes. Adolescence represents a critical period for psychological and behavioral development, during which aggressive behavior often correlates with future behavioral patterns (Moffitt, 1993). Examining aggression during this developmental stage enhances our understanding of its psychological and social determinants, thereby informing prevention and intervention strategies. For instance, studies suggest that the home environment, peer relationships, and school climate significantly influence adolescent aggression (Dishion and Patterson, 2016; Van Goozen et al., 2022).

Second, aggressive behavior detrimentally affects secondary school students' academic performance and social adjustment. It disrupts the classroom environment, impairs peers' learning experiences, and is associated with lower academic performance and a diminished sense of school belonging for the aggressor (Juvonen et al., 2011). Moreover, victims of aggressive behavior often experience psychological distress, including anxiety, depression, and low self-esteem (Hawker and Boulton, 2000).

Third, identifying the determinants of middle school students' aggressive behavior is crucial for designing effective interventions. Parent-child relationships and teacher caring behaviors have been extensively studied as key influences. Positive parent-child relationships and caring teacher behaviors are believed to mitigate adolescent aggression (Wentzel, 1997; Hoeve et al., 2009). A comprehensive investigation of these relationships can provide a scientific foundation for family education and school-based interventions aimed at preventing and reducing adolescent aggression.

### The effect of parent-child relationships on the aggressive behavior of middle school students

Parent-child relationships are fundamental to the psychological and behavioral development of middle school students (Branje et al., 2010; Pinquart, 2017). Studies indicate that positive parent-child relationships significantly reduce adolescent aggression, whereas poor relationships are strongly linked to aggressive behavior (Hoeve et al., 2009). The parent-child relationship involves not only parental emotional support and behavioral guidance but also communication patterns, conflict resolution strategies, and parenting approaches (Liu et al., 2022). These factors collectively shape adolescent behavior and mental wellbeing.

First, emotional support serves as a cornerstone of the parent-child relationship. Studies have found that parental emotional support and understanding enhance adolescents' self-esteem and sense of security, thereby mitigating aggressive behavior (Laursen and Collins, 2009). Adolescents who experience parental love and support are more inclined to adopt constructive coping strategies for stress and conflict, thereby reducing aggressive responses (Maccoby, 2000). Conversely, emotionally unsupportive home environments can contribute to adolescent loneliness and neglect, thereby heightening the risk of aggressive behavior (Patterson et al., 2015).

Second, family communication patterns and conflict resolution strategies significantly influence adolescent aggression. Open and effective communication enables adolescents to express their emotions and needs, thereby mitigating aggression stemming from misunderstandings and repressed emotions (Simpson, 2020). Studies indicate that frequent familial conflict and ineffective conflict resolution, including violence and verbal abuse, exacerbate adolescent aggression (Drummond et al., 2020; Zhou et al., 2022). Adolescents raised in such environments often imitate parental behavior and resort to aggression as a conflict resolution strategy (Akers and Jennings, 2015).

Furthermore, parenting strategies play a crucial role in shaping adolescent aggression. Authoritative parenting, characterized by high emotional support and clear behavioral expectations, is widely regarded as the most effective style for fostering adolescent development (Akers and Jennings, 2015). This parenting approach helps adolescents comprehend the consequences of their actions, fostering self-control, and responsibility, which in turn reduces aggressive behavior (Steinberg, 2001). Conversely, both authoritarian (high control, low support) and permissive (low control, low support) parenting styles are associated with heightened adolescent aggression (Kim and Crepaldi, 2021).

Based on the classic two-dimensional model proposed by Maccoby (2000), contemporary research on parenting typically categorizes parenting behavior into two fundamental dimensions: *responsiveness* and *demandingness* (Steinberg, 2001). *Responsiveness* refers to parents' emotional responsiveness and support for their children's needs, encompassing emotional warmth (e.g., showing care) and open communication (e.g., encouraging expression). *Demandingness* pertains to the enforcement of behavioral standards and supervision, including conflict resolution strategies (e.g., consensus-based discipline) and the granting of autonomy (e.g., negotiated rules). This study focused on emotional support and open communication within the responsiveness dimension, and constructive conflict resolution within the demandingness dimension—elements that collectively characterize authoritative parenting (Baumrind, 2013).

### The effects of teacher caring behavior on aggressive behavior in middle school students

Teacher caring behavior is pivotal in shaping the psychological development and behavioral regulation of secondary school students. Empirical research indicates that teacher caring behavior fosters students' sense of school belonging and self-esteem while significantly is mitigating aggressive tendencies (Ahn et al., 2021; Wentzel, 1997). Teacher caring behaviors encompass academic support, emotional care, and behavioral guidance, collectively fostering students' development of positive behavioral patterns and social competence (Pianta et al., 2003).

First, academic support from teachers can strengthen students' sense of achievement and self-efficacy, thereby reducing aggressive behaviors. Academic support extends beyond content instruction to include attentive guidance and assistance with students' learning difficulties (Reddy et al., 2003). When students perceive strong support and encouragement from their teachers, they are more likely to cultivate interest and confidence in learning, thereby minimizing aggressive responses induced by academic stress and perceived failure (Wentzel, 2002). Second, emotional support constitutes a fundamental aspect of teachers' caring behaviors. Empirical research suggests that teacher-student emotional connections play a crucial role in shaping students' emotional regulation and behavioral outcomes (Murray and Greenberg, 2000). Teachers' emotional care is demonstrated through understanding, respect, and attentiveness, fulfilling students' emotional needs while alleviating feelings of isolation and exclusion (Pianta et al., 2003). When students perceive emotional support from their teachers, they are more inclined to adopt constructive approaches to interpersonal conflicts, thereby reducing aggressive behavior (Hamre and Pianta, 2001).

Furthermore, teachers' behavioral guidance plays a crucial role in preventing and mitigating student aggression. Effective behavioral guidance encompasses well-defined behavioral norms, positive reinforcement strategies, and constructive disciplinary approaches (Baker et al., 2008). By setting explicit behavioral expectations and norms, teachers can facilitate students' understanding of the consequences of their actions while fostering self-control and responsibility (Jennings and Greenberg, 2009). Empirical research indicates that teachers can mitigate classroom conflict and violence while enhancing students' socialization through positive behavioral guidance (Osher et al., 2010).

The influence of teachers on students' behavior is exerted through multiple mechanisms (Wentzel, 2016). In addition to emotional support, effective behavior management strategies—such as establishing clear rules and providing timely intervention—are also essential for mitigating aggressive behavior (Gregory et al., 2010). The anti-bullying program developed by Olweus (1993) suggests that teachers must assume multiple roles, including supporter (providing emotional safety), supervisor (discouraging aggression), and arbiter (enforcing discipline). This study primarily examines the impact of supportive behaviors, while acknowledging the importance of other management strategies, which are identified as promising directions for future research.

### The relationship between parent-child relationship, professor caring behavior, and aggressive behavior in middle school students

Family and school are the two core ecosystems of adolescent growth (Sun et al., 2021), and their interaction has a profound impact on individual behavior development. Existing studies have shown that parent-child relationship and teacher caring behavior do not independently affect adolescent aggressive behavior, but there are complex synergistic or compensatory mechanisms (Li et al., 2021; Meehan et al., 2003; Zhu et al., 2025).

Teacher care may play an important compensatory role when family dysfunction occurs, such as high parent-child conflict and low emotional support. According to the risk-protection model, positive resources in the school environment (such as teacher support) are able to buffer the negative effects of family risk factors on adolescent development. For example, a study of adolescents from low-income families found that high levels of teacher care significantly reduced the incidence of aggressive behavior despite poor parent-child relationship quality (Fagan, 2020; Collins et al., 2017). This compensatory effect may be achieved through the following mechanisms: (1) Emotional support from teachers makes up for the lack of secure attachment in the family and meets the belonging needs of adolescents (Al-Yagon et al., 2016); (2) Teachers' positive behavior management strategies (such as positive reinforcement) provide alternative social learning paradigms and reduce opportunities for imitation of aggressive behavior (Khadka, 2024).

On the other hand, good parent-child relationship and high teacher care may form a synergistic protective effect. Social capital theory (Mikiewicz, 2021) points out that when family and school social resources are consistent, their promoting effects on adolescent development will be superimposed. Empirical studies have shown that in families with high parent-child affinity, teacher care has a stronger inhibitory effect on aggressive behavior (Heatly and Votruba-Drzal, 2017). This may be because positive communication patterns in the family (such as open-ended discussions) enhance adolescents' perception and ability to utilize school support (Medeiros et al., 2021). In addition, the cooperation between parents and teachers (such as home-school communication) can establish uniform behavioral norms and reduce the behavior disorder of adolescents in different situations (Marcu et al., 2019).

In summary, teacher caring behaviors influence middle school students' aggressive behaviors through several aspects such as academic support, emotional care, and behavioral guidance. Good caring teacher behavior can provide emotional support and behavioral guidance to help students adopt positive ways of coping with stress and conflict, thereby reducing aggressive behavior. Therefore, studying the effects of teachers' caring behaviors on secondary school students' aggressive behaviors not only helps to understand the mechanisms of their behavioral development, but also provides a scientific basis for school education and behavioral interventions.

While numerous studies have investigated the impact of caring teacher behaviors on students' aggressive tendencies, the majority have employed cross-sectional designs, limiting their ability to uncover dynamic relationships and causal mechanisms among the variables. Cross-lagged modeling is a statistical approach capable of simultaneously assessing interactions and lagged effects among variables, making it particularly suitable for examining the dynamic relationship between teacher caring behaviors and student aggression (Selig and Little, 2012). By collecting and analyzing data across multiple time points, this study sought to elucidate the lagged effects and interactions between teacher caring behaviors and middle school students' aggression, thereby offering theoretical insights and practical guidance for the prevention and intervention of adolescent aggression.

**Figure 1:**
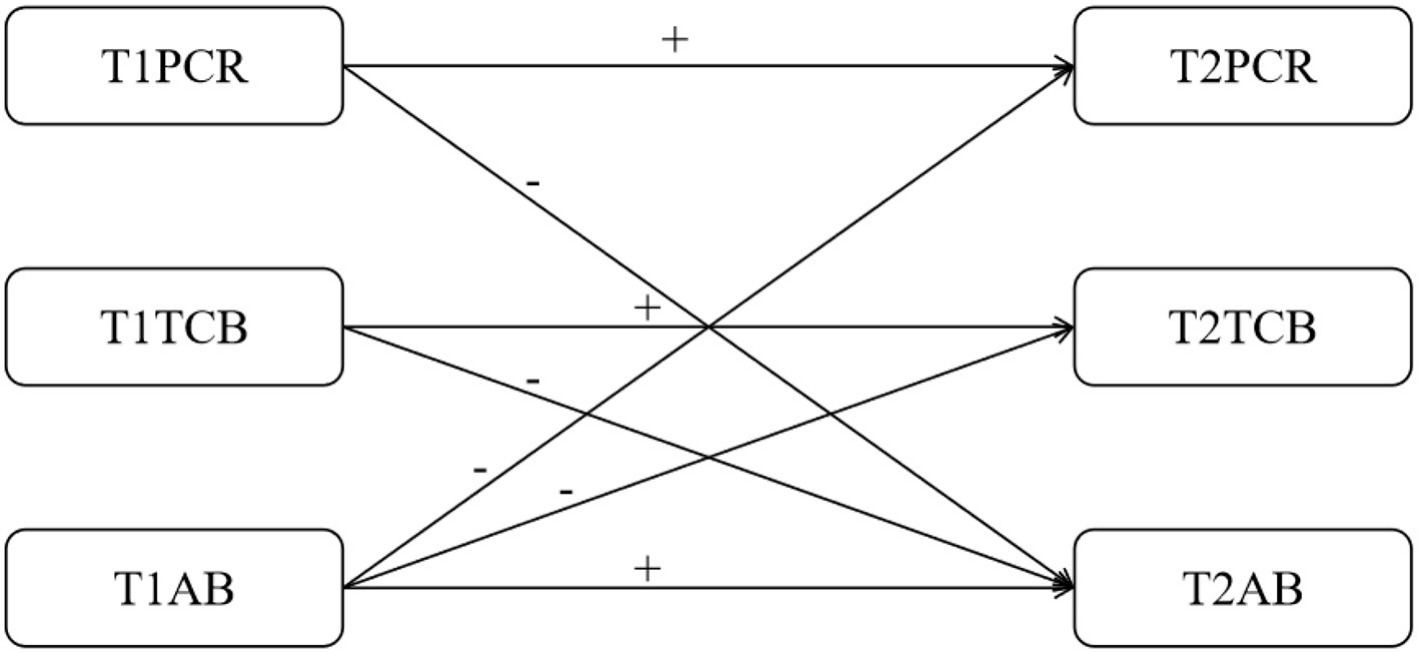
A hypothetical model of cross-lagged relationships between parental relationships, teacher caring behaviors, and aggression.

Based on the aforementioned content, this study proposes the following hypotheses, with the hypothesis model diagram shown in Figure 1: according to social control theory and social learning theory, this study proposes the following hypotheses: (1) There is a significant longitudinal association between parent-child relationships and aggressive behavior among middle school students, with early parent-child conflicts positively predicting later aggressive behavior, while parent-child cohesion negatively predicts aggressive behavior; (2) Teacher supportive behavior has a protective effect on aggressive behavior among middle school students, with higher levels of teacher support in early stages leading to fewer instances of aggressive behavior later on; (3) Aggressive behavior may also have a reverse impact on parent-child relationships and teacher supportive behavior, forming a bidirectional interaction path. By analyzing through cross-lagged models, this study aims to reveal the dynamic interaction mechanisms among the three factors, providing theoretical basis for campus psychological intervention.

## Method

### Research subjects and tools

Using convenience sampling, students from one high school and one middle school within Shandong Province were selected as participants. The initial measurement (T1) was conducted 2 weeks into the new semester, utilizing the Aggression Behavior Scale, Parent-Child Relationship Scale, and Teacher Care Scale. The second measurement (T2) took place at the end of the semester, employing the same measurement tools as T1. Both measurements were administered in class, with each session lasting ~45 min. The experiment was conducted by psychology graduate students with prior testing experience. A total of 964 participants took part in the initial measurement (T1), as shown in Table 1 while 897 participated in the subsequent measurement (T2), resulting in a dropout rate of 6.95%. There were 824 participants with complete data at both T1 and T2 time points, yielding an effective rate of 91.86%. In this study, 824 middle school students participated, including 443 males (53.8% of the total sample) and 381 females (46.2%). Participants were distributed across six grades: 135 first-year middle school students (16.4%), 132 second-year middle school students (16%), 143 third-year middle school students (17.4%), 121 first-year high school students (14.7%), 133 second-year high school students (16.1%), and 160 third-year high school students (19.4%). The educational attainment of fathers among the participants was distributed as follows: 239 had a primary education or lower (29% of the total sample), 228 had a junior high school education (27.7%), 189 had a senior high school education (22.9%), and 168 had a bachelor's degree or higher (20.4%). For mothers, the distribution was: 212 had a primary education or lower (25.7%), 234 had a junior high school education (28.4%), 199 had a senior high school education (24.2%), and 179 had a bachelor's degree or higher (21.7%). Among the participants, 434 were only children (52.7%) and 390 were not only children (47.3%). The geographical distribution included 278 from rural areas (33.7%), 262 from towns (31.8%), and 284 from urban areas (34.5%). The average age of the participants was 14.65 years, with a standard deviation of 2.27 years. This indicates that the sample covered a wide range of ages, encompassing students from middle school to high school grades. This research protocol was approved by the Ethics Committee of Jeonbuk National University (20240331JE346) and complies with the ethical standards of the 1964 Helsinki Declaration and its later amendments. All participants signed informed consent forms.

**Table 1:** Descriptive statistics of participants.

**Variable**	**Category**	**Frequency (*n*)**	**Percentage (%)**
Gender	Male	443	53.8
	Female	381	46.2
Grade level	7th Grade	135	16.4
	8th Grade	132	16
	9th Grade	143	17.4
	10th Grade	121	14.7
	11th Grade	133	16.1
	12th Grade	160	19.4
Father's educational level	Elementary school or below	239	29
	Middle school	228	27.7
	High school	189	22.9
	Bachelor's degree or above	168	20.4
Mother's educational level	Elementary school or below	212	25.7
	Middle school	234	28.4
	High school	199	24.2
	Bachelor's degree or above	179	21.7
Only child	Yes	434	52.7
	No	390	47.3
Place of residence	Rural	278	33.7
	Town	262	31.8
	Urban	284	34.5
Age		*M*	SD
		14.65	2.27

### Measuring tool

#### Aggressive behavior and anger measurement questionnaire

The Aggressive Behavior and Anger Measurement Questionnaire (ABAMQ) was adapted from the Chinese revision of the Buss-Perry Aggression Questionnaire (BPAQ), which has been widely validated in adolescent populations. This 20-item self-report measure assesses four theoretically derived dimensions of aggression: physical aggression (e.g., “I have threatened people I know”), anger (e.g., “I flare up quickly but get over it quickly”), hostility (e.g., “I am suspicious of overly friendly strangers”), and alternative aggression (e.g., “When frustrated, I express my anger indirectly”). Respondents rate each item on a 5-point Likert scale ranging from 1 (extremely uncharacteristic of me) to 5 (extremely characteristic of me). Psychometric evaluations have demonstrated excellent internal consistency for the full scale (α = 0.929), with subscale reliabilities ranging from 0.76 to 0.84. Confirmatory factor analysis supported the four-factor structure (χ^2^*/df* = 2.87, CFI = 0.93, TLI = 0.91, RMSEA = 0.06).

#### Parent-child relationship

The Parent-Child Relationship Scale (PCRS) is a multidimensional instrument assessing four key aspects of parent-adolescent interactions: (1) understanding and communication (e.g., “My parents try to understand my perspective”), (2) harshness and interference (e.g., “My parents criticize me excessively”), (3) love and respect (e.g., “My parents show affection toward me”), and (4) growth and tolerance (e.g., “My parents encourage my personal development”). The 20-item scale employs a 5-point Likert response format (1 = “not at all compliant” to 5 = “fully compliant”), with higher scores indicating more positive relationship quality. Psychometric evaluations have demonstrated excellent reliability, with the full scale showing a Cronbach's alpha of 0.964 in the current sample, and subscale alphas ranging from 0.84 to 0.92. Confirmatory factor analysis supported the theoretically-derived four-factor structure (χ^2^*/df* = 2.35, CFI = 0.95, TLI = 0.93, RMSEA = 0.05). Notably, the understanding/communication and love/respect subscales showed particularly strong associations with positive youth outcomes in previous studies. The PCRS's comprehensive assessment of both positive and negative relationship dimensions makes it particularly suitable for examining the complex dynamics between parenting behaviors and adolescent development in Chinese cultural contexts.

#### Teacher caring behavior

The Teacher Caring Behavior Scale (TCBS; Lei, 2014) was employed to assess students' perceptions of teacher support. This 18-item instrument measures three theoretically grounded dimensions of teacher care: (1) due diligence (e.g., “My teacher carefully checks my homework”), (2) support (e.g., “My teacher encourages me when I face difficulties”), and (3) inclusion (e.g., “My teacher makes sure all students participate in class activities”). Respondents rate each item on a 5-point Likert scale (1 = “not at all compliant” to 5 = “fully compliant”), with higher composite scores indicating more positive perceptions of teacher caring behaviors. Psychometric analyses revealed excellent internal consistency for the full scale (α = 0.961) in the current sample, with subscale reliabilities ranging from 0.86 to 0.91. Confirmatory factor analysis supported the three-factor structure (χ^2^*/df* = 2.18, CFI = 0.96, TLI = 0.95, RMSEA = 0.04). The inclusion dimension has shown particular relevance for predicting student engagement, while the support dimension has demonstrated strong associations with adolescent psychological wellbeing. The TCBS's multidimensional assessment of teacher care makes it particularly valuable for examining the protective role of teacher behaviors in adolescent development.

#### Statistical analysis

Data analysis was conducted using SPSS 21 and Amos 21. Descriptive statistics for the participants' demographic information were first computed using SPSS, followed by correlation analysis to examine the relationships among aggression, parent-child relationships, and teacher caring behaviors. Finally, a cross-lagged model analysis was conducted using Amos.

#### Common method bias test

Despite several measures, including standardized test administration in classrooms, assurances of anonymity and confidentiality, and restricting data use to academic research, there remains a potential risk of common method bias due to the self-reported nature of the questionnaire data. Following Podsakoff et al. (2003), a Harman's single-factor test was conducted on T1 and T2 measures of aggressive behavior, parent-child relationships, and teacher caring behaviors. The results indicated that 13 factors had eigenvalues >1, with the first factor accounting for only 10.86% of the variance, which is below the 40% threshold. Thus, it can be concluded that common method bias is not a concern in this study.

#### Related analysis

Table 2 presents the descriptive statistics and bivariate correlations among study variables across two time points (T1 and T2). At T1, aggressive behavior showed significant negative correlations with both parent-child relationship (*r* = −0.295, *p* < 0.01) and teacher caring behavior (*r* = −0.265, *p* < 0.01). These negative associations persisted at T2, with T1 parent-child relationship (*r* = −0.308, *p* < 0.01) and teacher caring behavior (*r* = −0.260, *p* < 0.01) both negatively predicting T2 aggressive behavior. Notably, T1 teacher caring behavior showed significant positive correlations with both T1 (*r* = 0.468, *p* < 0.01) and T2 (*r* = 0.225, *p* < 0.01) parent-child relationship, suggesting potential cross-system influences. All variables demonstrated acceptable variability (SDs ranging 0.890–1.182).

**Table 2:** Descriptive statistics and correlation analysis of variables.

**Variable**	***M***	**SD**	**T1 Aggressive behavior**	**T1 Parent-child relationship**	**T1 Teacher caring behavior**	**T2 Aggressive behavior**	**T2 Parent-child relationship**	**T2 Teacher caring behavior**
T1 Aggressive behavior	2.507	0.890	1					
T1 Parent-child relationship	2.918	1.109	−0.295^**^	1				
T1 Teacher caring behavior	2.731	1.182	−0.265^**^	0.468^**^	1			
T2 Aggressive Behavior	2.426	0.933	0.166^**^	−0.308^**^	−0.260^**^	1		
T2 Parent-child relationship	2.405	0.903	−0.014	0.048	0.053	−0.016	1	
T2 Teacher caring behavior	2.389	0.896	−0.012	0.120^**^	0.225^**^	−0.025	0.178^**^	1

^**^p < 0.01.

Table 3 presents the descriptive statistics of different gender groups and the results of correlation analysis between variables. In terms of aggressive behavior, the initial levels of boys (*M* = 2.518) and girls (*M* = 2.493) were similar at T1, but the decline of boys (Δ = 0.112) was slightly larger than that of girls (Δ = 0.043) at T2. Correlation analysis showed that both sexes showed temporal stability of aggressive behavior (boys *r* = 0.148, girls *r* = 0.188, *p*s < 0.01), but the cross-temporal correlation was stronger in girls, suggesting that their aggressive patterns may be more persistent.

**Table 3:** Descriptive statistics and correlation analysis of variables by gender.

**Gender**	**Variable**	***M***	**SD**	**T1 Aggressive behavior**	**T2 Aggressive behavior**	**T1 Parent-child relationship**	**T2 Parent-child relationship**	**T1 Teacher caring behavior**	**T2 Teacher caring behavior**
Male	T1 Aggressive behavior	2.518	0.878	1					
	T2 Aggressive behavior	2.406	0.937	0.148^**^	1				
	T1 Parent-child relationship	2.922	1.098	−0.272^**^	−0.278^**^	1			
	T2 Parent-child relationship	2.392	0.915	0.036	−0.041	0.039	1		
	T1 Teacher caring behavior	2.718	1.176	−0.269^**^	−0.288^**^	0.406^**^	0.091	1	
	T2 Teacher caring behavior	2.399	0.881	0.009	−0.026	0.068	0.142^**^	0.192^**^	1
Female	T1 Aggressive behavior	2.493	0.905	1					
	T2 Aggressive behavior	2.450	0.929	0.188^**^	1				
	T1 Parent-child relationship	2.913	1.123	−0.320^**^	−0.342^**^	1			
	T2 Parent-child relationship	2.420	0.889	−0.072	0.014	0.058	1		
	T1 Teacher caring behavior	2.745	1.191	−0.261^**^	−0.228^**^	0.539^**^	0.007	1	
	T2 Teacher caring behavior	2.378	0.914	−0.034	−0.022	0.177^**^	0.220^**^	0.262^**^	1

^**^p < 0.01.

The negative correlation between parent-child relationship and aggressive behavior at T2 was significantly stronger in girls (*r* = −0.342) than in boys (*r* = −0.278), which may reflect that girls are more sensitive to family emotional environment. Notably, the negative correlation between teacher caring and aggressive behavior showed a cross-time point effect in boys (T1 teacher caring and T2 aggressive behavior *r* = −0.288), while it mainly showed a concurrent correlation in girls (T1 teacher caring and T1 aggressive behavior *r* = −0.261).

Gender differences were also found in inter-system correlations: girls showed a stronger parent-child relationship with teacher caring at the same time (*r* = 0.539 at T1 vs. *r* = 0.406 for boys), while boys showed a more temporal stability of teacher caring behavior (*r* = 0.192 at T1–T2 vs. *r* = 0.177 for girls). These differences provide important implications for sex-specific intervention strategies.

#### Cross-lagged model test

**Figure 2:**
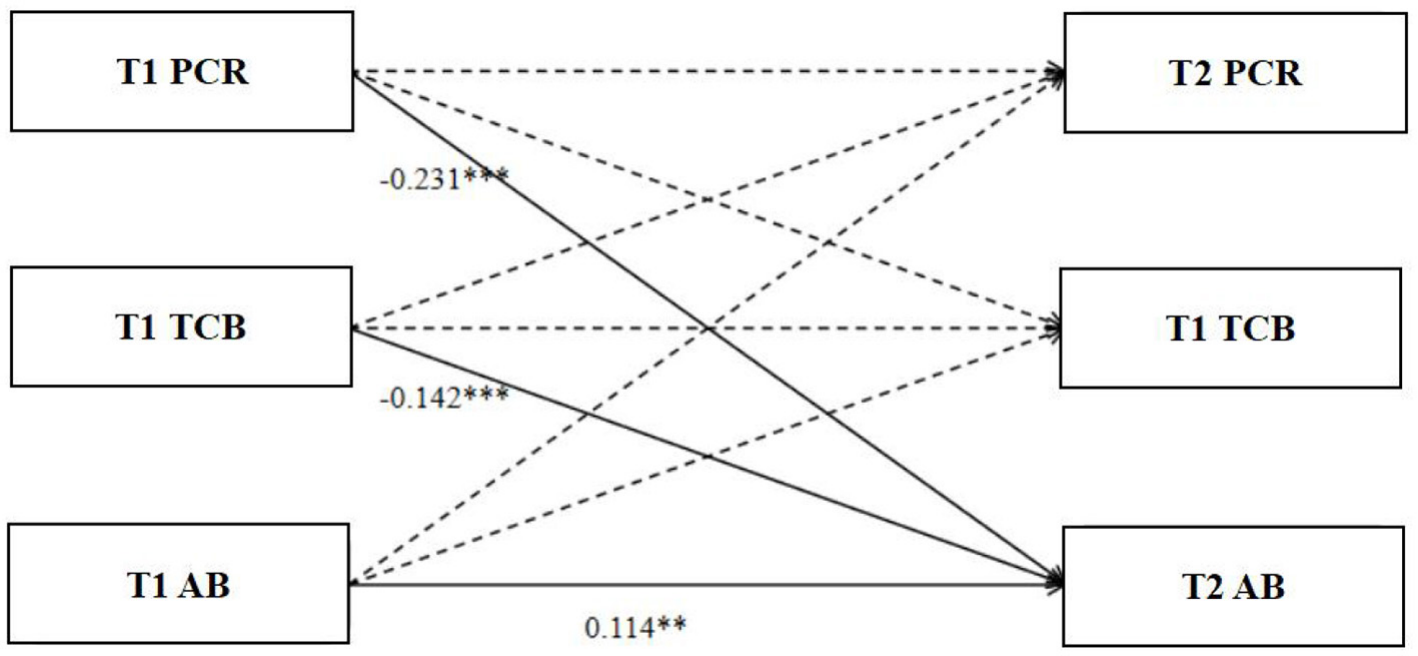
Cross-lagged relationships between parent-child relationships, caring teacher behaviors, and aggression (CFI = 0.978, AGFI = 0.922, RMSEA = 0.048, 90% CI[0.043, 0.052], χ^2^*/df* = 2.861, *p* < 0.05). **p < 0.01, ***p < 0.001.

As shown in Figure 2, T1 parent-child relationships significantly and positively influenced T2 teacher caring behavior (β = 0.097, *SE* = 0.031, *t* = 2.63, *p* = 0.009), but did not have a significant effect on T2 parent-child relationships (β = 0.037, *SE* = 0.031, *t* = 1.018, *p* = 0.309). T1 parent-child relationships had a significant negative effect on T2 aggressive behavior (β = −0.231, *SE* = 0.03, *t* = −6.437, *p* < 0.001), suggesting that a positive parent-child relationship can help reduce future aggressive behavior. T1 teacher caring behavior did not significantly influence T2 parent-child relationships (β = 0.038, *SE* = 0.029, *t* = 1.045, *p* = 0.296) or T2 teacher caring behavior (β = 0.05, *SE* = 0.029, *t* = 1.355, *p* = 0.175), but had a significant negative effect on T2 aggressive behavior (β = −0.142, *SE* = 0.028, *t* = −3.981, *p* < 0.001), suggesting that teacher caring behavior also plays a role in reducing future aggressive behavior. T1 aggressive behavior had no significant effect on T2 parent-child relationships (β = 0.031, *SE* = 0.037, *t* = 0.84, *p* = 0.401) or T2 teacher caring behavior (β = 0.064, *SE* = 0.036, *t* = 1.7, *p* = 0.089) but had a significant positive effect on T2 aggressive behavior (β = 0.114, *SE* = 0.036, *t* = 3.165, *p* = 0.002), suggesting that aggressive behavior remains relatively stable between the two time points. Overall, the findings underscore the crucial role of parent-child relationships and teacher caring behavior in mitigating aggressive behavior among middle school students.

## Discussion

This study aimed to investigate the dynamic effects of parent-child relationships and teacher caring behavior on aggressive behavior among middle school students. Using a cross-lagged model, we analyzed the interactions among parent-child relationships, teacher caring behavior, and aggression across multiple time points. The results indicated that both parent-child relationships and teacher caring behavior significantly influenced middle school students' aggressive behavior. First, parent-child relationships had a significant impact on aggressive behavior. This finding aligns with previous research, highlighting the central role of parent-child relationships in adolescent behavioral regulation (Hoeve et al., 2009). Positive parent-child relationships are often characterized by high levels of emotional support and effective communication, which facilitate the development of emotional regulation and social skills, ultimately reducing aggressive behavior (Laursen and Collins, 2009). Furthermore, teacher caring behavior has a significant negative effect on aggressive behavior. This indicates that teachers' care and support can mitigate students' aggressive behavior. This finding underscores the crucial role of teachers in students' psychological development, particularly in providing emotional support and behavioral guidance (Wentzel, 1997). Teacher caring behavior fosters a positive sense of belonging in school and promotes constructive behavioral patterns through positive reinforcement and effective disciplinary strategies (Pianta et al., 2003). Additionally, the study identified a high degree of stability in aggressive behavior across the two time points. This result is consistent with existing literature, reinforcing the persistence and stability of aggressive behavior during adolescence (Loeber and Hay, 1997; Naseem and Munaf, 2022). However, the relatively low stability of parent-child relationships and teacher caring behavior suggests that these relationships may be susceptible to external influences and changes.

### The influence of parent-child relationships on aggressive behavior in middle school students

In longitudinal studies, the parent-child relationship has been found to exert a significant negative effect on aggressive behavior in middle school students, a phenomenon widely supported and explained in psychological literature. As a key factor in the socialization process of adolescents, a positive parent-child relationship is typically characterized by high levels of emotional support, effective communication, and consistent behavioral norms. These elements collectively contribute to reducing aggressive behavior in adolescents (Laursen and Collins, 2009).

First, emotional support is a fundamental component of the parent-child relationship. Research indicates that parental emotional support enhances adolescents' emotional regulation and social adjustment, thereby reducing aggressive behavior (Maccoby, 2000). When adolescents perceive care and understanding from their parents, they are more likely to develop positive emotion regulation strategies, which, in turn, mitigate aggressive reactions triggered by emotional distress (Eisenberg et al., 2001). Beyond fostering psychological security, emotional support also equips adolescents with the resilience to respond constructively to stress and challenges (Collins and Laursen, 2004).

Secondly, effective communication plays a crucial role in the parent-child relationship. Open and constructive communication enables adolescents to express their emotions and needs, thereby reducing aggressive behaviors stemming from suppressed emotions and misunderstandings (Grych and Fincham, 1990). Through meaningful interactions with their parents, adolescents develop essential conflict resolution and problem-solving skills, which help mitigate aggressive responses in interpersonal conflicts (Frazer et al., 2021). Research indicates that frequent and effective family communication is significantly associated with lower levels of aggressive behavior in adolescents (Cummings et al., 2000).

In addition, consistent behavioral norms and discipline are fundamental aspects of the parent-child relationship. Authoritative parenting, which combines high levels of emotional support with clear behavioral expectations, is widely regarded as the most conducive to adolescent development (Frazer et al., 2021; Kim and Crepaldi, 2021). This parenting style helps adolescents understand the consequences of their actions and fosters self-control and a sense of responsibility, ultimately reducing aggressive behavior (Steinberg, 2001). Conversely, inconsistent discipline or excessively harsh parenting approaches may contribute to increased defiant and aggressive behaviors in adolescents (Patterson et al., 2015).

Longitudinal studies offer the advantage of elucidating the dynamic processes and causal mechanisms underlying the relationship between parent-child interactions and adolescent aggression. By collecting and analyzing data across multiple time points, these studies can more accurately capture the long-term effects of parent-child relationships on adolescent aggressive behavior (Selig and Little, 2012). For instance, a meta-analysis by Hoeve et al. (2009) demonstrated that the negative effects of emotional support deficits and inadequate behavioral guidance in parent-child relationships on adolescent aggression were significant across multiple time points. This finding further underscores the critical role of parent-child relationships in shaping adolescent behavioral development.

In summary, the significant negative effect of parent-child relationships on aggression in middle school students has been well substantiated by longitudinal studies. Positive parent-child relationships foster adolescents' emotional regulation and social adjustment by providing emotional support, facilitating effective communication, and establishing consistent behavioral norms, thereby mitigating aggressive behavior. This finding offers a crucial theoretical foundation and practical implications for family education and behavioral interventions.

### Significant negative effects of teacher caring behavior on aggressive behavior of middle school students

Longitudinal studies have consistently demonstrated a significant negative association between teacher caring behaviors and aggressive behavior in middle school students, a finding widely supported in psychological research. Teacher caring behaviors—such as emotional support, academic assistance, and behavioral guidance—are crucial for students' mental wellbeing and behavioral self-regulation (Ahn et al., 2021). These supportive behaviors contribute to a reduction in aggressive tendencies among middle school students through multiple psychological and social mechanisms.

First, teachers' emotional support fosters students' sense of school belonging and self-esteem, which in turn helps mitigate aggressive behavior. Research indicates that students who perceive their teachers as caring and understanding exhibit significantly improved emotional regulation and social adjustment (Hamre and Pianta, 2001). Such emotional support creates a psychologically safe environment, encouraging students to adopt constructive coping strategies instead of resorting to aggression when facing stress and challenges (Murray and Greenberg, 2000). For instance, students who receive strong emotional support from teachers are more likely to form positive peer relationships, thereby decreasing aggression stemming from social frustration (Baker et al., 2008).

Second, teachers' academic support strengthens students' sense of achievement and self-efficacy, thereby reducing aggressive behavior. Academic support extends beyond content delivery to include personalized attention and assistance for students facing learning difficulties (Reddy et al., 2003). When students receive academic support and encouragement from teachers, they are more likely to develop an interest in learning and gain confidence, which helps mitigate aggressive responses caused by academic stress and perceived failure (Wentzel, 2002). Such academic support fosters a positive self-concept, which in turn reduces aggressive behavior (Pianta et al., 2003).

Furthermore, teachers' behavioral guidance is crucial in preventing and mitigating student aggression. Effective behavioral guidance encompasses clearly defined norms, positive reinforcement, and constructive disciplinary strategies (Jennings and Greenberg, 2009). By setting explicit behavioral expectations and norms, teachers help students comprehend the consequences of their actions and cultivate self-control and responsibility, thereby decreasing aggressive behavior (Osher et al., 2010). Research indicates that positive behavioral guidance enables teachers to reduce classroom conflict and violence while fostering students' socialization (Pianta and Ryan, 2002).

By collecting and analyzing data across multiple time points, this study more accurately captured the long-term impact of teacher caring behaviors on student aggression (Selig and Little, 2012). For instance, Hamre and Pianta (2001) demonstrated that early positive teacher-student relationships significantly predicted students' behavioral outcomes in later school years, reinforcing the crucial role of teacher caring behaviors in shaping student conduct.

### Stabilizing effects of aggressive behavior in middle school students

In this longitudinal study, middle school students exhibited a high degree of stability in aggressive behavior, and this stability can be attributed to various mechanisms, including individual traits, family environment, social influences, and the self-reinforcement of established behavioral patterns.

First, individual traits are crucial determinants of the stability of aggressive behavior. Research has shown that certain personality traits, such as impulsivity and hostility, are significantly associated with aggressive behavior (Caspi et al., 2005). These traits exhibit a high degree of stability throughout an individual's development, contributing to the persistence of aggressive behavior. For example, adolescents with high impulsivity traits are more likely to engage in aggressive behavior when faced with conflict, and this behavioral pattern demonstrates significant stability over time (Loeber and Hay, 1997; Naseem and Munaf, 2022).

Second, the family environment plays a critical role in the stability of aggressive behavior. Parenting styles, parent-child relationships, and the overall family climate influence the development of adolescent behaviors (Patterson et al., 2015). For example, a home environment that lacks emotional support and effective communication may increase adolescents' propensity for aggressive behavior when faced with stress. Research has found that negative patterns of interaction in the family, such as frequent conflict and inconsistent disciplinary measures, contribute to the persistence of aggressive behavior (Cummings et al., 2000).

In addition, social influence plays a crucial role in the stability of aggressive behavior. Adolescents' peer relationships and school environments strongly influence their behavioral development (Costello et al., 2020; Dishion et al., 1996). Within peer groups, aggressive behaviors may become normalized and replicated, leading to their maintenance and reinforcement over time. This social reinforcement mechanism allows aggressive behavior to exhibit long-term stability (Huesmann et al., 1984; Siann, 2024).

Self-reinforcement of behavioral patterns serves as a crucial mechanism in explaining the stability of aggressive behavior. According to social learning theory, an individual's behavioral patterns are sustained by self-reinforcement and environmental feedback (Akers and Jennings, 2015). Once aggressive behavior develops, individuals may gain reinforcement through outcomes such as exerting control over others or resolving conflicts, further strengthening this behavioral pattern. Research has shown that early manifestations of aggressive behavior are strong predictors of future aggression, and this self-reinforcing mechanism demonstrates long-term persistence (Huesmann et al., 2009).

### Research shortcomings and prospects

Although this study revealed the dynamic relationship between middle school students' aggressive behavior and both parent-child and teacher caring behaviors through a longitudinal design and cross-lagged model, several limitations persist and should be addressed in future research. First, the data for this study were primarily derived from self-report questionnaires, which may have introduced social desirability effects and reporting bias (Podsakoff et al., 2003). Future research could incorporate multi-source data collection methods, such as teacher assessments, peer assessments, and observational techniques, to enhance data objectivity and accuracy (Achenbach et al., 1987).

Second, the sample for this study consisted of middle school students from a specific region, resulting in a relatively homogeneous sample in terms of geographic and cultural background, potentially limiting the generalizability of the findings (Henrich et al., 2010). Future research should consider conducting similar studies in diverse cultural contexts and geographical settings to assess the cross-cultural applicability and generalizability of these findings (Bornstein, 2017).

Although the longitudinal design of this study revealed dynamic relationships between variables, it's relatively short time interval may have limited its ability to capture the long-term effects of parent-child relationships and caring teacher behaviors on aggressive behavior (Selig and Little, 2012). Future research could extend the time span of data collection and implement longitudinal studies over extended periods to gain a more comprehensive understanding of the long-term changes and trajectories of these relationships (Moffitt, 1993).

Additionally, this study did not extensively examine the effects of other potential mediating and moderating variables, such as peer relationships, school climate, and individual psychological traits, on aggressive behavior (Dishion et al., 1994). Future research could explore the mediating and moderating roles of these variables in the relationship between parent-child interactions, teacher caring behaviors, and aggression by developing more sophisticated models to reveal a more comprehensive understanding of behavioral development mechanisms (Pretorius, 2020). In addition, this study focused on emotional support behaviors of teachers and did not systematically examine other important dimensions such as discipline management. In addition, the measurement of parenting behavior focuses on the responsive aspect, and the manipulation of the demanding dimension is relatively limited. These limitations suggest that future studies should use more comprehensive measurement tools.

In summary, although this study contributed to understanding the dynamic relationship between middle school students' aggressive behavior, parent-child relationships, and teachers' caring behaviors, several limitations remain. Future studies should enhance data collection methods, increase sample diversity, extend the time span, incorporate latent variables, and improve causality validation to advance the understanding of secondary school students' aggressive behaviors and their influencing factors. These improvements will help establish a stronger empirical foundation for educational and psychological interventions.

## Conclusion

Using a longitudinal design and cross-lagged modeling, this study provides insight into the dynamic relationship between aggression and parent-child and teacher caring behaviors among middle school students. The results of the study indicated that both good parent-child relationships and caring teacher behaviors significantly and negatively predicted aggression in middle school students, and that this effect had a lagged effect over time. Specifically, emotional support and effective communication in the parent-child relationship, as well as emotional support, academic support, and behavioral guidance in teacher caring behaviors, can help to reduce aggressive responses in middle school students. Additionally, the study found that middle school students' aggressive behaviors showed a high degree of stability over time, further emphasizing the importance of early intervention.

The findings of this study provide an important theoretical basis for understanding the formation mechanisms of aggression in middle school students and provide empirical support for educational practices and behavioral interventions. By strengthening parent-child relationships at home and caring teacher behaviors at school, secondary school students' aggressive behaviors can be effectively reduced to promote their mental health and social adaptability. However, there are some limitations of the study, and future research should improve on sample diversity, data collection methods, and exploration of potential variables to further deepen the understanding of aggression and its influencing factors among middle school students. Overall, this study emphasized the important role of the family and school environments in the behavioral development of secondary school students, and called for concerted efforts by multiple parties to promote the healthy growth of adolescents.

## Data Availability

The raw data supporting the conclusions of this article will be made available by the authors, without undue reservation.
